# Elastomeric Pain Pumps for Scoliosis Surgery

**DOI:** 10.7759/cureus.4042

**Published:** 2019-02-11

**Authors:** Hilda H Kriel, David Yngve

**Affiliations:** 1 Orthopedics, Baylor Scott & White Mclane Children's Medical Center, Temple, USA; 2 Orthopaedics, The University of Texas Medical Branch, Galveston, USA

**Keywords:** scoliosis, multimodal analgesia, elastomeric pain pumps, surgery, pediatric surgery, pain management, adolescent idiopathic scoliosis

## Abstract

Objective

Postoperative pain management following scoliosis surgery has traditionally relied on intravenous opioids. The objective of this study was to evaluate the effect of elastomeric pain pumps.

Methods

A retrospective chart review of 81 adolescent patients who underwent scoliosis surgery in a seven-year period was performed. Patients were divided into three groups as the practice changed: (1) patient-controlled analgesia group (12 patients) who used intravenous (IV) opioids with oral opioids; (2) elastomeric pain pump group (28 patients) with the added use of bupivacaine; and (3) multimodal pain pump group (41 patients) with the added use of gabapentin and methocarbamol. Endpoints were analyzed for length of stay in the hospital, infection rate, and gastrointestinal retention.

Results

The length of stay in the elastomeric pain pump group was 3.1 days shorter than in the patient-controlled analgesia group (*P* = 0.004). The length of stay in the multimodal group was 3.9 days shorter than in the patient-controlled analgesia group (*P* = 0.001). The incidence of prolonged postoperative bowel retention decreased significantly from 25% to 18% to 2% (*P* = 0.03).

Conclusions

To our knowledge, this is the first study on the use of elastomeric pain pumps in conjunction with multimodal pain medication following scoliosis surgery. The use of elastomeric pain pumps was associated with clinically and statistically significant improvements in the postoperative course. The addition of methocarbamol and gabapentin was associated with a trend toward further improvements.

## Introduction

Multimodal analgesia has been shown to be effective in reducing opioid use in adult spine and joint arthroplasty patients, but little data is available on multimodal analgesia in adolescent spine patients [1-2]. Traditionally, postoperative pain management for adolescent scoliosis patients has relied on patient-controlled intravenous and oral opioids. However, opioids are associated with side effects that include respiratory depression, pruritus, constipation, nausea, vomiting, and sedation, which can lead to delayed recovery time and patient discomfort.

Shorter hospital stays reduce exposure to infection, allow earlier return to function and normality for the family, and decrease hospital costs. It was hypothesized that the use of elastomeric pain pumps improves the postoperative course of adolescent scoliosis patients.

## Materials and methods

A retrospective chart review of 81 adolescent patients (average age: 13 years, range: 2 to 18 years) who underwent scoliosis surgery by a single orthopedic surgeon in a seven-year period from March 2007 to December 2014 was performed. Excluded were surgeries for adjustment of growing rods and anterior surgeries. Patients were divided into three groups as practice changed. The patient-controlled analgesic (PCA) group (12 patients) used intravenous opioids with oral opioids; the elastomeric pain pump (EPP) group (28 patients) added the use of three-day elastomeric pain pumps containing 270 ml of 0.125% or 0.25% bupivacaine; and the multimodal pain pump (MPP) group (41 patients) added multimodal pain management, including gabapentin and methocarbamol. Oral gabapentin was given preoperatively at 15 mg/kg and was continued at 5 mg/kg three times daily for seven days. Methocarbamol 500 mg was given orally every six hours if the patient weighed over 33 kg. Endpoints were analyzed for length of stay (LOS) in the hospital, infection rate, and postoperative gastrointestinal retention, defined as the inability to tolerate a soft diet at 48 hours postoperatively. The elastomeric pain pumps were placed subfascially and were removed on postoperative day three. Patients received Duramorph intrathecally and intravenous ketorolac at the surgeon's discretion. Patient diet was advanced based on clinical improvement and with the auscultation of bowel sounds.

There were no formal hospital efforts to reduce LOS during the study. There was no change in physical therapy protocols. Some surgeons might become more comfortable with shorter LOS as their practice matures, but this is difficult to ascertain. Implant material was changed from stainless steel to titanium later in the study, affecting the MPP group. Also, the surgical blood loss was decreasing as the surgeon’s practice matured. Gentamycin was added to the bone graft later in the study as well.

Each of the variables was described using means and standard deviations, or proportions, for continuous and categorical variables. LOS, treatment group, and gender were assessed using analysis of variance (ANOVA) or the *t*-test and association with age by using correlation. The categorical outcome variables of infection, postoperative gastric retention, and neurogenic etiology of the scoliosis were tested for association with each treatment group and with gender using chi-square and Fisher’s exact test. Association with age was tested using the *t*-test. Pairwise comparisons of LOS between the three treatment groups were carried out using Tukey’s honestly significant difference (HSD) test.

## Results

Patient demographics are shown in Table 1. There was no significant difference between the three groups with regard to demographic data. There were similar ratios between male and female and between idiopathic or neuromuscular etiology. The use of elastomeric pain pumps did not increase the rate of infection. There was a significant difference in the rate of infection between the three groups (Fisher’s exact value: 0.0046). The PCA group had the highest rate of infection (Table 2). This finding may be independent of the type of analgesia since the patients later in the series had gentamicin mixed with allograft bone, which has been shown to reduce deep postoperative infection [3]. Another significant difference was noted in the rate of postoperative gastrointestinal retention, with the PCA group having the highest rate of retention noted postoperatively. The MPP group showed the lowest rate of postoperative gastrointestinal retention at 2%. Rates were 25%, 17%, and 2% in the three groups (Table 2).

**Table 1:** Demographic data in three groups PCA = patient-controlled analgesic group (opioids only), EPP = elastomeric pain pump group (opioids and elastomeric pain pump), MPP = multimodal pain pump group (opioids, elastomeric pain pumps, gabapentin, and methocarbamol)

	PCA (n = 12)	EPP (n = 28)	MPP (n = 41)	P-values
Gender				0.48
Male	2	5	13	
Female	10	23	28	
Age	13.4 ± 2.7	13.5 ± 2.6	13.8 ± 2.4	0.79
Neuromuscular				1
No	8	19	27	
Yes	4	9	14	

**Table 2:** Postoperative factors in the three groups PCA = patient-controlled analgesic group (opioids only), EPP = elastomeric pain pump group (opioids and elastomeric pain pump), MPP = multimodal pain pump group (opioids, elastomeric pain pumps, gabapentin, and methocarbamol)

	PCA	EPP	MPP	P-values
Infection				0.005
Yes	3 (25%)	2 (7.1%)	0 (0%)	
No	9 (75%)	26 (92.9%)	41 (100%)	
Postoperative gastrointestinal retention				0.014
Yes	3 (25%)	5 (17.9%)	1 (2.4%)	
No	9 (75%)	23 (82.1%)	40 (97.6%)	
Length of stay (days)	8.2 ± 6.4	5.1 ± 1.2	4.3 ± 1.3	0.00094

The results also showed a significant difference in the length of stay for the three groups of patients, with the MPP group having a shorter length of stay (Table 2). On average, the length of stay in the EPP group was 3.1 days shorter than in the PCA group (*P* = 0.004). Also, the length of stay in the MPP group was 3.9 days shorter than in the PCA group (*P* = 0.001). The length of stay in the MPP group was 0.8 days less than the EPP group (*P* = 0.8).

Overall, there were significant and clinically important differences between the PCA group and the elastomeric pain pump groups (EPP and MPP). Differences between the two elastomeric pain pump groups, with and without gabapentin and methocarbamol, were small but trended in a favorable direction in the three variables studied.

## Discussion

Postoperative pain control affects patient comfort, the ability to ambulate and eat, and, consequently, the LOS. The addition of elastomeric pain pumps and other multimodal medications reduced the LOS of our patients to values comparable to, or lower than, those cited in the literature. Sources cite as typical five days for adolescent idiopathic scoliosis patients and seven days for neuromuscular scoliosis patients [1,4]. While our study combined idiopathic and neuromuscular scoliosis, our LOS significantly decreased over time. This trend has also been demonstrated in other studies. One found a decrease in LOS from 6.3 days in 2000 to 5.4 days in 2009 [5]. Another showed that in patients with neuromuscular scoliosis, the LOS decreased from 9.21 days in 2002 to 6.70 days in 2011 [6]. An additional study showed that an accelerated discharge protocol of early ambulation and multimodal pain medication resulted in a decrease from five days to 3.7 days in patients with adolescent idiopathic scoliosis, with a 22% decrease in hospital charges during the study period [7].

A study comparing epidural analgesia to intravenous and oral opiates following adolescent scoliosis surgery showed improved pain scores and reduced side effects in the epidural group. However, complications and the effect on LOS were not discussed [8]. Another study compared continuous infusion of local anesthetics (CILA) from elastomeric pain pumps with epidural analgesia. Those modalities were shown to have equivalent pain control. The elastomeric pain pump showed a decreased rate and duration of urinary catheter placement, which is a benefit. The LOS in these patients was not discussed [9]. Another study on scoliosis patients compared CILA with elastomeric pain pumps in conjunction with intrathecal morphine injection with PCA and oral opioids. The elastomeric pain pump patients relied less on opioids in the first 24 hours postoperatively but did not have reduced pain or opioid use on day two, when they began ambulating [10].

Because of its effectiveness in neuropathic pain, gabapentin was evaluated in the setting of scoliosis surgery in combination with PCA and oral opioids. Gabapentin has been shown to be opioid-sparing in the adult population in both single preoperative dosing and postoperatively, but it has not been extensively studied in children. One randomized controlled trial showed a decreased amount of postoperative morphine use and lower pain scores with the addition of preoperative and postoperative gabapentin [11]. However, the addition of only a single preoperative dose of gabapentin showed no effect on postoperative opioid usage, pain scores, or side effects [12]. Our results showed decreased LOS with elastomeric pain pumps alone and when combined with gabapentin and methocarbamol.

The mechanism of action of methocarbamol is centrally acting muscle relaxation with some mildly sedating effects. It has been used in postoperative pain management in breast reconstruction. One study in children compared open versus minimally invasive pectus excavatum repair. The combination of methocarbamol, nonsteroidal anti-inflammatory drugs (NSAIDs), and narcotics reduced hospital costs [13].

A criticism of elastomeric pain pumps is the concern for increased postoperative infection. Our data showed a decreasing trend of infection during the time when elastomeric pain pumps were introduced. Bupivacaine at the concentrations used (0.125% to 0.25%) has been shown to inhibit the growth of bacteria [14]. Our later cases did also have the addition of gentamicin to the allograft bone, as well as povidone-iodine wound irrigation.

The second most common cause for readmission after wound complications is gastrointestinal disturbance [15]. Our data showed a significant decrease in gastrointestinal retention with the use of elastomeric pain pumps both with and without multimodal pain medication. The rate of postoperative gastrointestinal retention decreased with each of our groups through time, from 25% to 17% to 2%.

This study has limitations. It was retrospective, and so we had to rely on available documentation. Also, the three groups were not equivalent in size and the study periods were not concurrent. However, our LOS improvement compared favorably with other reports. Finally, the cases were done by a single surgeon, which decreases some variability.

## Conclusions

To our knowledge, this is the first study on the use of elastomeric pain pumps as part of multimodal therapy following scoliosis surgery. The use of pain pumps was associated with clinically and statistically improved postoperative outcomes. The addition of methocarbamol and gabapentin was associated with a trend towards further improvements.
